# Safety and feasibility of continuous ketamine infusion for analgosedation in medical and cardiac ICU patients who received mechanical ventilation support: A retrospective cohort study

**DOI:** 10.1371/journal.pone.0274865

**Published:** 2022-09-22

**Authors:** Hohyung Jung, Jihye Lee, Hyun Young Ahn, Jeong Hoon Yang, Gee Young Suh, Ryoung-Eun Ko, Chi Ryang Chung

**Affiliations:** 1 Department of Critical Care Medicine, Samsung Medical Center, Sungkyunkwan University School of Medicine, Seoul, Republic of Korea; 2 Department of Pharmaceutical Services, Samsung Medical Center, Sungkyunkwan University School of Medicine, Seoul, Republic of Korea; 3 Division of Cardiology, Department of Medicine, Samsung Medical Center, Sungkyunkwan University School of Medicine, Seoul, Republic of Korea; 4 Division of Pulmonary and Critical Care Medicine, Department of Medicine, Samsung Medical Center, Sungkyunkwan University, Seoul, Republic of Korea; 5 Department of Medicine, Samsung Medical Center, Sungkyunkwan University, Seoul, Republic of Korea; National Taiwan University Hospital, TAIWAN

## Abstract

**Purpose:**

To assess the effect of continuous ketamine administration in patients admitted to medical and cardiac intensive care units (ICUs) and received mechanical ventilation support.

**Methods:**

We conducted a retrospective cohort study between March 2012 and June 2020 at an academy-affiliated tertiary hospital. Adult patients who received mechanical ventilation support for over 24 h and continuous ketamine infusion for at least 8 h were included. The primary outcome was immediate hemodynamic safety after continuous ketamine infusion. The secondary outcomes included immediate delirium, pain, and use of sedation.

**Results:**

Of all 12,534 medical and cardiac ICU patients, 564 were eligible for the analysis. Ketamine was used for 33.3 (19.0–67.5) h and the median continuous infusion dose was 0.11 (0.06–0.23) mcg/kg/h. Of all patients, 469 (83.2%) received continuous ketamine infusion concomitant with analgosedation. Blood pressure and vasopressor inotropic scores did not change after continuous ketamine infusion. Heart rate decreased significantly from 106.9 (91.4–120.9) at 8 h before ketamine initiation to 99.8% (83.9–114.4) at 24 h after ketamine initiation. In addition, the respiratory rate decreased from 21.7 (18.6–25.4) at 8 h before ketamine initiation to 20.1 (17.0–23.0) at 24 h after ketamine initiation. Overall opioid usage was significantly reduced: 3.0 (0.0–6.0) mcg/kg/h as fentanyl equivalent dose at 8 h before ketamine initiation to 1.0 (0.0–4.1) mcg/kg/h as fentanyl equivalent dose at 24 h post-ketamine initiation. However, the use of sedatives and antipsychotic medications did not decrease. In addition, ketamine did not increase the incidence of delirium within 24 h after ketamine infusion.

**Conclusion:**

Ketamine may be a safe and feasible analgesic for medical and cardiac ICU patients who received mechanical ventilation support as an opioid-sparing agent without adverse hemodynamic effects.

## Introduction

Analgesia and sedation are essential elements of care for mechanically ventilated patients in the intensive care unit (ICU). Although the 2018 clinical practice guidelines for adult patients in the ICU recommended light sedation and early mobilization, some mechanically ventilated patients require deep sedation during the early phase of acute respiratory distress syndrome management [1, 2]. For deep sedation, intravenous administration of opioids, benzodiazepines, and propofol is often preferred in the ICU. However, their use is associated with complications, including dependence, delirium, and adverse hemodynamic changes.

Ketamine is a non-competitive N-methyl-D-aspartate receptor antagonist that blocks glutamate and induces both analgesia and sedation [3]. A recent meta-analysis reported an increasing trend of ketamine use in mechanically ventilated patients and showed that ketamine might be used as a sedative-sparing agent [4]. In addition, several randomized control trials demonstrated the clinical benefits of continuous ketamine infusion in critically ill patients [5, 6]. The respiratory drive is preserved with a chronotropic effect on the cardiovascular system at therapeutic doses. However, most previous studies were conducted with small sample sizes in trauma, postoperative, or cerebral ischemia patients. Therefore, the usefulness of continuous ketamine infusion was not supported in the medical and cardiac ICUs, where many mechanically ventilated patients are often admitted.

This study assessed serial hemodynamic, pain, agitation, and sedation trends; analgesic and sedative interventions; and the incidence of delirium before and after continuous ketamine infusion in patients who were admitted to medical and cardiac ICUs and received mechanical ventilation.

## Materials and methods

### Study design

**Fig 1:**
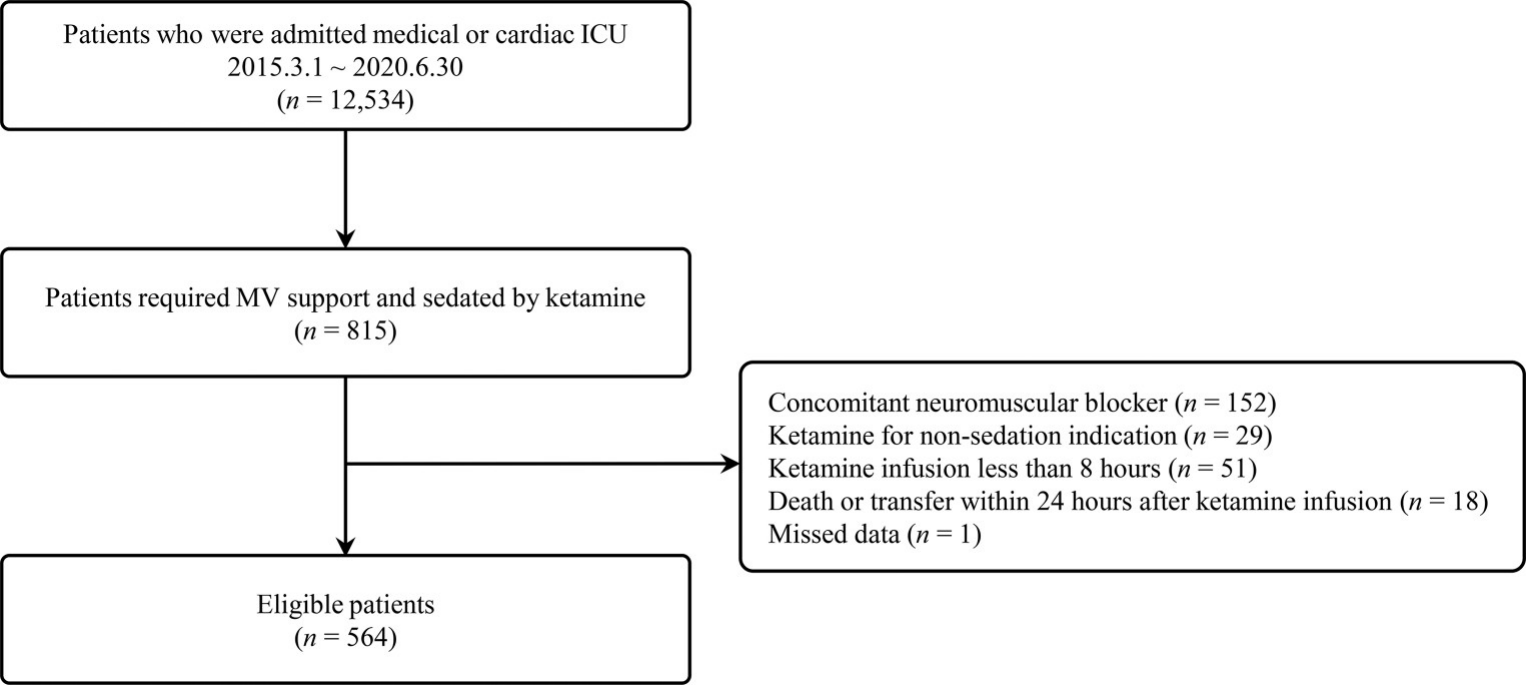
Flow diagram. ICU, intensive care unit; MV, mechanical ventilator.

We conducted a retrospective cohort study between March 2012 and June 2020 at the Samsung Medical Center (a 1,989-bed tertiary referral hospital with tertiary-level ICUs) in Seoul, South Korea. We screened all patients aged 18 years or older who were admitted to medical and cardiac ICUs over 24 h. The admission units were decided according to main problems and underlying co-morbidities. Patients who received mechanical ventilation via tracheal intubation for over 24 h and continuous ketamine infusion for at least 8 h were included in the study. A total of 815 patients received continuous ketamine infusion during the study period. Patients who used concomitant neuromuscular blockers (n = 152) and ketamine for the non-sedation indication (n = 29), used ketamine for less than 8 h (n = 51), who were deceased or transferred within 24 h after the start of continuous ketamine infusion (n = 18), and had missing data (n = 1) were excluded. The remaining 564 eligible patients were analyzed (Fig 1).

The Institutional Review Board of the Samsung Medical Center approved this study and waived the requirement for informed consent because of the observational nature of the study. In addition, the patients’ information was anonymized and de-identified before analysis.

### Setting

The clinical practice guidelines published by the Society of Critical Care Medicine were adopted for general intensive care [1]. The institution did not have a protocol for patient selection for continuous ketamine infusion. In general, ketamine was added to conventional analgosedation infusions to optimize pain or sedation in patients who have failed conventional analgosedation or experienced adverse effects secondary to conventional analgosedation. The initiation of ketamine administration was based on the physician’s discretion. When ketamine was started, a dosing protocol recommended a starting infusion of 2.0 mcg/kg/min to be titrated by 0.5 mcg/kg/min up to a maximum rate of 7.0 mcg/kg/min. According to the 2018 clinical practice guideline, we adjusted other opioids and sedatives to target a Richmond Agitation and Sedation Scale of -2 to +1 [1].

### Data collection and clinical outcomes

Over the study period, all patients admitted to the medical or cardiac ICUs were prospectively registered. For this study, the data were supplemented with a retrospective review of electronic medical records. Demographic data, sequential organ failure assessment (SOFA) score, simplified acute physiology score 3, comorbidity, and diagnosis were collected at ICU admission. Serial data, including vital signs and the dose of vasopressors and inotropes were collected as average values before and after ketamine initiation. Serial data collection included 8 h before, time zero signifying ketamine initiation, and 8 h, 16 h, and 24 h post-ketamine initiation. vasoactive inotropic score (VIS) was calculated as: dopamine dose (μg/kg/min) + dobutamine dose (μg/kg/min) + 100 × epinephrine dose (μg/kg/min) + 10 × milrinone dose (μg/kg/min) + 10,000 × vasopressin dose (unit/kg/min) + 100 × norepinephrine dose (μg/kg/min) [7]. Due to the light sedation strategy, even in patients in controlled ventilation, the respiratory rate of the patient and the setting of respiratory rate on a mechanical ventilator may differ. Serial data were extracted using our institution’s data repository (Clinical Data Warehouse Darwin-C, Samsung Medical Center, Seoul, Korea). Pain was evaluated using a critical care pain observation tool or numeric rating scale, and delirium was evaluated using the Richmond Agitation Sedation Scale and confusion assessment method for the ICU. Cardiac problems were defined as patients initially diagnosed with acute myocardial infarction, cardiogenic shock, complex arrhythmia, acute congestive heart failure with respiratory failure and/or requiring hemodynamic support, unstable angina, cardiac arrest, cardiac tamponade, dissecting aortic aneurysms, or complete heart block.

The primary outcome was immediate hemodynamic safety after continuous ketamine infusion. Hemodynamic safety was assessed by serial mean arterial blood pressure, heart rate, and respiratory rate changes. The secondary outcomes included immediate delirium, pain, and use of sedation.

### Statistical analyses

Continuous variables were expressed as medians and interquartile ranges (IQRs) and examined using the Mann–Whitney U-test. Categorical variables were presented as numbers and percentages and analyzed using the Chi-square test or Fisher’s exact test. Subgroup analysis was performed according to primary diagnosis, admitted ICU, and shock. The serial changes in the clinical variables were calculated using the Jonckheere–Terpstra test. All data were analyzed using SPSS version 22 (IBM Corp., Armonk, NY, USA).

## Results

### Study population

The baseline characteristics of the patients are shown in Table 1. There were 386 (68.4%) men, and the median age was 68 (59–76) years. Of all patients, 362 (64.2%) patients received controlled ventilation, and 202 (35.8%) received supported ventilation. Malignancies (49.8%) and cardiovascular diseases (16.0%) were the most frequent comorbidities. Of all eligible patients, 486 (86.2%) were admitted to the medical ICU, and 78 (13.8%) were admitted to the cardiac ICU. The median initial SOFA score was 9 (6–12). The most common diagnosis at ICU admission was respiratory failure (57.4%), followed by sepsis (19.5%) and cardiovascular disease (16.7%). During the ICU stay, 89 (15.8%) patients received continuous renal replacement therapy, and 57 (10.1%) received extracorporeal membrane oxygenation. The median length of mechanical ventilation support day was 6.7 (3.1–13.4). Overall, 181 patients (32.1%) died in the hospital. After comparing medical ICU patients with cardiac ICU patients, the clinical characteristics are presented in S1 Table.

**Table 1:** Clinical characteristics and clinical outcomes of patients who received continuous ketamine infusion.

Variables	Patients who received ketamine infusion (N = 564)
Sex, male	386 (68.4)
Age, year	68 (59–76)
Body mass index, kg/m^2^	22.9 (20.3–25.4)
Co-morbidity	
Cardiovascular disease	90 (16.0)
Respiratory disease	61 (10.8)
Gastrointestinal/hepatobiliary disease	30 (5.3)
Malignancies	281 (49.8)
Chronic neurological disease	16 (2.8)
ICU location	
Medical ICU	486 (86.2)
Cardiac ICU	78 (13.8)
Severity score at ICU admission	
Initial SOFA score	9 (6–12)
Initial SAPS 3	62 (50–73)
Primary diagnosis at ICU admission	
Respiratory failure	324 (57.4)
Sepsis	110 (19.5)
Cardiovascular problems	94 (16.7)
Gastrointestinal/hepatobiliary problems	28 (5.0)
Neurologic problems	3 (0.5)
Others	5 (0.9)
Organ support during ICU stay	
Continuous renal replacement therapy	89 (15.8)
Extracorporeal membrane oxygenation	57 (10.1)
Vasopressor	392 (69.5)
Total length of MV support, days	6.7 (3.1–13.4)
Clinical outcomes	
Length of ICU stay, days	10.0 (5.4–19.1)
In-ICU mortality	181 (32.1)

Data are presented as median and interquartile ranges or as numbers (%) of patients.

ICU, intensive care unit; SOFA, sequential organ failure assessment; SAPS, Simplified Acute Physiology Score; CAM-ICU, confusion assessment method for the intensive care unit; MV, mechanical ventilation.

### Continuous ketamine infusion

The median VIS at the start of continuous ketamine infusion was 4.0 (0.0–18.2). Detailed analgosedation infusions at the start of ketamine administration are shown in Table 2. Remifentanil was the most commonly used opioid (35.1%, 0.10 [0.05–0.20] mcg/kg/min), and dexmedetomidine was the most commonly used sedative (9.7%, 0.3 [0.2–0.6] mcg/kg/h). During ICU stay, ketamine was used at 33.3 (19.0–67.5) h. The median continuous infusion dose was 6.7 (3.5–14.1) mg/kg/min. Of all patients, 469 (83.2%) received continuous ketamine infusion concomitant with analgosedation.

**Table 2:** Characteristics at the start of continuous ketamine infusion.

Variables	Patients who received ketamine infusion (N = 564)
Vasoactive Inotropic Score	4.0 (0.0–18.2)
Lactic acid, mmol/L	2.5 (1.6–4.8)
History of cardiopulmonary arrest during hospital stay	54 (9.6)
Ventilator mode	
Controlled ventilation	335 (59.4)
Supported ventilation	229 (40.6)
Opioids*	1.3 (0.0–5.2)
Fentanyl	
Used patients	67 (11.9)
Dose, mcg/kg/hr	3.0 (2.0–5.0)
Remifentanil	
Used patients	198 (35.1)
Dose, mcg/kg/min	0.10 (0.05–0.20)
Hydromorphone	
Used patients	61 (10.8)
Dose, mg/hr	1.9 (0.7–3.0)
Morphine	
Used patients	3 (0.5)
Dose, mg/hr	5.0 (4.0–5.5)
Sedatives	
Dexmedetomidine	
Used patients	55 (9.7)
Dose, mcg/kg/hr	0.3 (0.2–0.6)
Midazolam	
Used patients	17 (3.0)
Dose, mg/hr	0.10 (0.05–0.20)
Propofol	
Used patients	29 (5.1)
Dose, mcg/kg/min	15.0 (10.0–30.0)
Total use of ketamine infusion	
Starting dose, μg/kg/min	2.0 (2.0–2.0)
Duration, hr	33.3 (19.0–67.5)
Total dose, mg/kg/min	6.7 (3.5–14.1)
Concomitant use of analgesic and sedative	469 (83.2)

Data are presented as median and interquartile ranges or as numbers (%) of patients.

*Overall opioid usage was calculated at the fentanyl equivalent dose.

### Before and after continuous ketamine infusion

**Fig 2:**
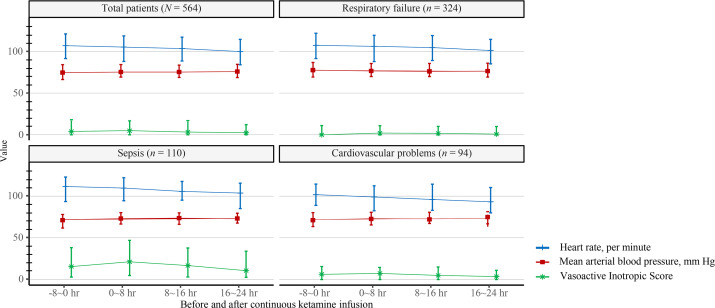
Hemodynamic trends during continuous ketamine infusion.

Of the 564 patients, blood pressure and VIS did not change before and after continuous ketamine infusion (Fig 2, Table 3). Heart rate decreased significantly from 106.9 (91.4–120.9) at 8 h before ketamine initiation to 99.8 (83.9–114.4) at 24 h after ketamine initiation. In addition, the respiratory rate decreased significantly from 21.7 (18.6–25.4) at 8 h before ketamine initiation to 20.1 (17.0–23.0) at 24 h after ketamine initiation.

**Table 3:** Hemodynamic trends during continuous ketamine infusion according to the diagnosis at ICU admission.

	0~8 hr before ketamine infusion	0~8 hr after ketamine infusion	8~16 hr after ketamine infusion	16~24 hr after ketamine infusion	P for trend
Total patients (N = 564)					
Systolic blood pressure, mm Hg	110 (96–129)	111 (98–127)	112 (99–127)	114 (99–129)	0.034
Diastolic blood pressure, mm Hg	57.8 (51.3–65.3)	58.5 (53.1–65.7)	58.3 (52.9–64.6)	58.1 (52.8–65.1)	0.652
Mean arterial blood pressure, mm Hg	74.5 (66.2–84.1)	75.2 (69.1–84.2)	75.2 (68.8–83.5)	75.8 (68.6–84.5)	0.156
Heart rate, per minute	106.9 (91.4–120.9)	105.2 (87.9–118.6)	103.4 (88.5–117.0)	99.8 (83.9–114.4)	< 0.001
Respiratory rate, per minute	21.7 (18.6–25.4)	20.8 (18.1–24.0)	20.3 (17.3–23.7)	20.1 (17.0–23.3)	< 0.001
Vasoactive Inotropic Score	4.0 (0.0–17.0)	4.9 (0.0–17.4)	3.3 (0.0–15.8)	2.5 (0.0–12.5)	0.491
Respiratory failure (N = 324)					
Systolic blood pressure, mm Hg	116 (101–134)	115 (100–129)	115 (102–131)	118 (101–131)	0.811
Diastolic blood pressure, mm Hg	58.9 (53.4–66.8)	58.5 (53.3–65.4)	58.3 (52.9–64.7)	58.0 (52.9–64.6)	0.121
Mean arterial blood pressure, mm Hg	77.5 (69.3–86.7)	76.5 (69.9–85.4)	76.0 (69.9–85.6)	76.3 (69.2–85.8)	0.354
Heart rate, per minute	107.2 (91.4–121.8)	106.0 (87.8–119.3)	104.5 (89.1–119.0)	101.0 (85.1–114.4)	< 0.001
Respiratory rate, per minute	21.4 (18.7–25.2)	21.0 (18.5–24.1)	20.6 (17.8–23.7)	20.2 (17.7–23.5)	0.001
Vasoactive Inotropic Score	0.0 (0.0–11.0)	2.0 (0.0–10.9)	1.7 (0.0–10.0)	0.8 (0.0–9.9)	0.907
Sepsis (N = 110)					
Systolic blood pressure, mm Hg	102 (89–118)	105 (93–118)	108 (97–117)	108 (97–120)	0.034
Diastolic blood pressure, mm Hg	55.4 (47.2–59.9)	57.3 (50.9–62.2)	57.4 (52.3–62.0)	56.5 (51.2–63.2)	0.043
Mean arterial blood pressure, mm Hg	70.0 (61.7–78.0)	73.1 (66.6–80.2)	74.8 (66.2–79.9)	73.2 (67.6–79.6)	0.044
Heart rate, per minute	111.6 (93.5–122.9)	109.7 (94.4–122.0)	105.7 (95.2–117.6)	103.7 (85.0–115.6)	0.002
Respiratory rate, per minute	23.6 (19.6–27.2)	21.2 (19.6–24.6)	21.4 (18.9–24.9)	21.2 (19.1–24.0)	< 0.001
Vasoactive Inotropic Score	15.6 (2.9–38.2)	21.3 (4.8–47.0)	16.8 (3.0–37.9)	10.7 (2.7–34.0)	0.500
Cardiovascular problems (N = 94)					
Systolic blood pressure, mm Hg	101 (86–118)	101 (88–118)	102 (90–120)	106 (92–121)	0.193
Diastolic blood pressure, mm Hg	56.8 (51.7–66.2)	59.2 (54.6–68.2)	59.6 (53.2–68.0)	60.0 (53.6–67.3)	0.359
Mean arterial blood pressure, mm Hg	68.8 (63.5–80.2)	73.2 (65.4–80.6)	72.1 (67.3–80.6)	74.9 (66.8–81.3)	0.041
Heart rate, per minute	101.8 (88.9–114.5)	99.0 (82.5–112.4)	96.0 (83.0–114.3)	93.2 (80.0–110.3)	0.051
Respiratory rate, per minute	20.4 (17.2–24.1)	19.5 (17.1–22.4)	19.2 (16.4–22.0)	18.3 (15.7–21.1)	0.001
Vasoactive Inotropic Score	6.3 (0.0–16.2)	7.4 (0.0–15.3)	5 (0.0–14.5)	3.6 (0.0–11.1)	0.192

Data are presented as median interquartile ranges.

Subgroup analyses were also performed to investigate hemodynamic changes after continuous ketamine infusion according to the diagnosis at ICU admission. In patients who were admitted for respiratory failure (n = 324), blood pressure and VIS were not changed before and after continuous ketamine infusion, while heart rate and respiratory rate were decreased significantly: 107.2 (91.4–121.8) at 8 h before ketamine initiation to 101.0 (85.1–114.4) at 24 h post-ketamine initiation for heart rate, and 21.4 (18.7–25.2) at 8 h before ketamine initiation to 20.2 (17.7–23.5) at 24 h after ketamine initiation for respiratory rate. The most surprising aspect of the data was in patients who were admitted with sepsis and cardiovascular problems. In patients who were admitted for sepsis (n = 110), arterial blood pressure was increased significantly after continuous ketamine infusion without increased VIS score: 70.0 (61.7–78.0) at 8 h before ketamine initiation to 73.2 (67.6–79.6) at 24 h after ketamine initiation for mean arterial blood pressure. Similar to patients with respiratory failure, heart rate and respiratory rate were significantly decreased. In addition, in patients who were admitted for cardiovascular problems (n = 94), arterial blood pressure also increased significantly after continuous ketamine infusion without increased VIS score, and heart rate and respiratory rate were significantly decreased.

The trends in pain, agitation, sedation, and delirium are shown in Table 4. No significant changes were observed in the prior- and post-ketamine initiation. The trends in pain, agitation, sedation, and delirium are evaluated according to clinical subgroups and presented in S2 Table. There were no significant changes prior- and post ketamine initiation whether in medical or cardiac ICU, sepsis, and shock. The analgesic and sedative interventions are presented in Table 5. Overall opioid usage was significantly reduced: 3.0 (0.0–6.0) mcg/kg/h as fentanyl equivalent dose at 8 h before ketamine initiation to 1.0 (0.0–4.1) mcg/kg/h as fentanyl equivalent dose in 24 h post-ketamine initiation. The use of remifentanil (p for trend = 0.010) and hydromorphone (p for trend = 0.010) were markedly reduced. However, the use of sedatives and antipsychotic medications did not decrease.

**Table 4:** Pain, agitation, and sedation trends during continuous ketamine infusion.

Total (N = 564)	0~8 hr before ketamine infusion	0~8 hr after ketamine infusion	8~16 hr after ketamine infusion	16~24 hr after ketamine infusion	P for trend
RASS					
-2 ~ 0	195 (34.6)	223 (39.5)	230 (40.8)	211 (37.4)	0.497
< -2 or > 0	248 (44.0)	235 (41.7)	209 (37.1)	218 (38.7)	0.174
-4 and -5	100 (17.7)	102 (18.1)	114 (20.2)	115 (20.4)	0.042
CAM-ICU					
Positive	349 (61.9)	363 (64.4)	326 (57.8)	314 (55.7)	0.602
Negative	89 (15.8)	88 (15.6)	102 (18.1)	107 (19.0)	0.174
Critical care pain observation tool					
≤ 2	453 (90.4)	477 (93.1)	477 (94.1)	468 (93.2)	0.200
Numeric rating scale					
≤ 3	37 (90.0)	26 (86.7)	27 (90.0)	30 (100.0)	0.368

Data are presented as numbers (%) of patients.

RASS, Richmond agitation sedation scale; CAM-ICU, confusion assessment method for the intensive care unit.

**Table 5:** Trends of analgesic and sedative interventions during continuous ketamine infusion.

Total (N = 564)	0~8 hr before ketamine infusion	0~8 hr after ketamine infusion	8~16 hr after ketamine infusion	16~24 hr after ketamine infusion	P for trend
Opioids*	3.0 (0.0–6.0)	1.5 (0.0–4.5)	1.2 (0.0–4.0)	1.0 (0.0–4.1)	<0.001
Fentanyl					
Used patients	90 (19.2)	95 (20.3)	98 (20.9)	87 (18.6)	0.800
Dose, mcg/kg/hr	3.0 (2.0–4.3)	2.3 (1.2–4.0)	2.0 (1.1–3.9)	2.0 (1.2–4.0)	0.839
Remifentanil					
Used patients	247 (52.7)	215 (45.8)	186 (39.7)	170 (36.2)	0.010
Dose, mcg/kg/min	0.10 (0.05–0.17)	0.09 (0.05–0.17)	0.10 (0.05–0.17)	0.10 (0.05–0.17)	< 0.001
Hydromorphone					
Used patients	59 (12.6)	86 (18.3)	87 (18.6)	93 (19.8)	0.010
Dose, mg/hr	1.6 (0.8–2.4)	1.1 (0.5–2.0)	1.1 (0.5–2.5)	1.5 (0.6–2.6)	0.005
Morphine					
Used patients	2 (0.4)	3 (0.6)	3 (0.6)	3 (0.6)	0.225
Dose, mg/hr	3.7 (2.6–4.9)	8.6 (7.3–9.0)	7.9 (6.9–8.9)	6.0 (3.1–8.0)	0.684
Sedatives					
Dexmedetomidine					
Used patients	82 (14.5)	61 (10.8)	62 (11.0)	69 (12.2)	0.800
Dose, mcg/kg/hr	0.31 (0.21–0.48)	0.21 (0.13–0.38)	0.21 (0.15–0.40)	0.20 (0.15–0.32)	0.379
Midazolam					
Used patients	22 (3.9)	21 (3.7)	21 (3.7)	23 (4.1)	0.684
Dose, mg/hr	0.09 (0.04–0.19)	0.06 (0.02–0.16)	0.06 (0.03–0.12)	0.07 (0.04–0.16)	0.893
Propofol					
Used patients	43 (7.6)	41 (7.3)	42 (7.4)	39 (6.9)	0.200
Dose, mcg/kg/min	22.5 (14.3–34.4)	9.8 (2.3–15.8)	13.6 (5.4–24.7)	18.7 (10.6–29.8)	0.748
Antipsychotic medication**	77 (13.7)	84 (14.9)	79 (14.0)	73 (12.9)	0.497

Data are presented as median and interquartile ranges or as numbers (%) of patients.

*Over all opioid usage was calculated at the fentanyl equivalent dose (mcg/kg/hr).

**Antipsychotic medication included olanzapine, haloperidol, and quetiapine.

## Discussion

This study evaluated serial hemodynamic, pain, agitation, sedation trends, and analgesic and sedative interventions before and after continuous ketamine infusion. Heart rate and respiratory rate decreased significantly without changing the mean blood pressure and VIS score. The trends in pain, agitation, sedation, and delirium did not change before and after continuous ketamine infusion. In analgesic and sedative interventions, a significant reduction in opioid use was observed, while sedatives and antipsychotic medications used similar prior- and post-continuous ketamine infusion.

Continuous ketamine infusion has been shown to reduce opioid requirements in patients with mechanical ventilation. Opioids are the cornerstone of pain management in critically ill patients. However, high cumulative doses of opioid use might be associated with opioid dependence, addiction, and hormonal changes [8]. Yaffe et al. analyzed 2,595 ICU survivors and reported that 12.2% of patients used opioids at hospital discharge, and 4.4% of survivors still used opioids up to 48 months of follow-up [9]. Academia and colleagues reviewed a total of 71 mechanically ventilated patients and reported that more than a third of opioid-naïve patients were discharged with large amounts of opioids after an ICU stay [10]. Therefore, the 2018 clinical practice guidelines recommend the use of non-opioid analgesics as adjunctive pain medications. Several studies of ketamine infusion in ICU patients have revealed that the use of low-dose ketamine as an adjunct to opioid therapy may help reduce opioid consumption, especially in surgical patients [11–13]. The study also found that continuous ketamine infusion reduced immediate opioid consumption in mechanically ventilated medical ICU patients. The present study suggests that continuous ketamine infusion might be helpful for non-opioid analgesics in critically ill patients. Recent meta-analysis for evaluation the impact of ketamine on opioids and sedatives consumption in critically ill patients also found similar results [14]. Some studies found that ketamine infusion reduced the use of benzodiazepine in critically ill patients [6, 15]. However, this study did not show a decrease in sedative requirements for patients on mechanical ventilation. Due to the small number of patients receiving sedatives, the effect of ketamine infusion on sedative requirement may not be fully evaluated in this study.

To date, the usefulness of ketamine infusion in sepsis is known, with several pieces of evidence. Recent studies on ketamine infusion have reported its anti-inflammatory effects. Sun et al. investigated the protective effect of ketamine in vivo and showed that ketamine suppressed the production of tumor necrosis factor-alpha and interleukin-6 via inhibition of nuclear factor-κB in the intestine [16]. In addition, ketamine has been shown to reduce leukocyte recruitment, downregulate iNOS and cyclooxygenase-2, and upregulate anti-inflammatory markers such as heme oxygenase-1 [17, 18]. In clinical practice, ketamine infusion has been reported to increase catecholamine levels, which may be associated with increased sympathetic activity and blood pressure [19, 20]. In contrast, several studies have suggested that ketamine reduces left ventricular systemic and diastolic function in patients with ischemic heart disease or children [21, 22]. A small study by Jakobsen et al. showed a decreased cardiac index after ketamine infusion in patients with heart failure despite an increase in mean arterial pressure and systemic vascular resistance [21]. In this study, mean arterial blood pressure tended to increase after continuous ketamine infusion in patients with sepsis and cardiovascular problems (p = 0.044 and p = 0.041, respectively; Fig 2). Further research should be undertaken to investigate the long-term trend of hemodynamic changes after continuous ketamine infusion in patients with sepsis and cardiovascular problems.

More recently, studies have emerged that offer contradictory findings about the association between ketamine and delirium. A recent randomized controlled trial of patients undergoing cardiac surgery with cardiopulmonary bypass reported that intraoperative ketamine-based anesthesia significantly reduced postoperative delirium compared to propofol-based anesthesia [23]. In addition, a meta-analysis of the effects of ketamine on mechanically ventilated patients also reported a lower incidence of delirium in patients with ketamine than in those without ketamine [4]. Meanwhile, a secondary analysis study of a large ICU cohort of 925 patients reported that ketamine in critically ill patients may increase the risk of ICU delirium (adjusted odds ratio 5.60; 95% confidence interval, 1.09–29.15) [24]. However, some studies have reported that ketamine did not affect the incidence of delirium [25, 26]. In this study, we also found that ketamine did not affect the incidence of delirium within 24 h after ketamine infusion in medical or cardiac critically ill patients. However, this result is limited by short-term observations. Considering that ketamine was associated with hallucinations, sleep disturbances, and nightmares, more research with long-term observation after ketamine infusion is needed to clarify the association between ketamine and delirium.

In this study, 324 patients with respiratory failure were included, and these patients showed a reduced respiratory rate compared to that before continuous ketamine infusion. A possible explanation for this might be that the patients became more sedated after ketamine infusion. Ketamine has favorable characteristics for patients with respiratory failure, including maintenance of respiratory drive and airway reflexes and bronchodilation [27, 28]. However, a recent randomized control trial showed that ketamine infusion was not associated with improvement in ventilator variables associated with bronchospasm [29]. The effect of ketamine on mechanically ventilated patients has been extended during the coronavirus disease 2019 (COVID-19) pandemic due to the long duration of mechanical ventilation and the possibility of other sedative shortage to keep acute respiratory failure patients on mechanical ventilatory support [30, 31]. Several comments on analgesia and sedation strategies in mechanically ventilated patients with COVID-19 recommend ketamine as an alternative drug for sedation [31, 32]. Experience with ketamine infusion in patients with COVID-19 may help to better understand the impact of ketamine on mechanically ventilated patients.

Although this study provides additional information on continuous ketamine infusion in mechanically ventilated patients, several limitations should be acknowledged. First, this was a retrospective cohort study conducted in a single referral hospital. Although general ICU management was conducted according to general guidelines, the generalizability of our findings to other settings may be limited. In addition, vital signs and medications can be assessed by reviewing an electrical medical record. Multi-center prospective studies are needed to clarify the effects of ketamine infusion in medical or cardiac patients. Second, we did not include long-term serial data on hemodynamic changes, pain, agitation, and sedation. A prospective study with long-term serial monitoring data is needed to confirm the safety and usefulness of continuous ketamine infusion in critically ill mechanically ventilated patients. Third, this study was not specifically designed to evaluate detailed clinical information including inflammatory markers, and the results of echocardiography which might present immediate effect of ketamine infusion. Finally, we did not collect data on the adverse effects of ketamine, including anxiety, nightmares, sleep disturbance, increased salivation, and frequent suction. However, we found that ketamine did not increase the incidence of delirium within 24 h after ketamine infusion. Further well-designed prospective studies for evaluation of feasibility of ketamine infusion in medical and cardiac ICU patients should include detailed clinical information.

## Conclusion

After ketamine infusion, heart rate and respiratory rate decreased significantly without changing the mean blood pressure and VIS score. In addition, a significant reduction in opioid use was observed. However, the incidence of delirium did not significantly increase within 24 h after ketamine infusion. This finding highlights that ketamine may be a safe and feasible analgesic for medical and cardiac ICU patients who received mechanical ventilation support.

## Supporting information

S1 Table Clinical characteristics and clinical outcomes of patients who received continuous ketamine infusion according to admitted ICU. (DOCX)

S2 Table Pain, agitation, and sedation trends during continuous ketamine infusion according to clinical subgroups. (DOCX)

## Abbreviations

ICU: intensive care unit
SOFA: sequential organ failure assessment
VIS: vasoactive inotropic score
COVID-19: coronavirus disease 2019
