# Supplementary material for: Safety and feasibility of continuous ketamine infusion for analgosedation in medical and cardiac ICU patients who received mechanical ventilation support: A retrospective cohort study
Source: PLoS One. 2022 Sep 22;17(9):e0274865. doi: 10.1371/journal.pone.0274865 (PMC9499237; doi:10.1371/journal.pone.0274865)
Supplement: S1 Table — (DOCX) [file pone.0274865.s001.docx]

**Supplementary Table 1.** Clinical characteristics and clinical outcomes of patients who received continuous ketamine infusion according to admitted ICU

| Variables | Medical ICU  (n=486) | Cardiac ICU  (n=78) | P |
| --- | --- | --- | --- |
| Sex, male | 326 (67.1) | 60 (76.9) | 0.108 |
| Age, year | 66.0 (57.0 – 75.0) | 68.0 (60.0 – 75.0) | 0.209 |
| Body mass index, kg/m^2^ | 22.7 (20.1 – 25.2) | 23.8 (21.5 – 27.3) | 0.018 |
| Co-morbidity |  |  | <0.001 |
| Cardiovascular disease | 37 (7.6) | 53 (67.9) |  |
| Respiratory disease | 57 (11.7) | 4 (5.1) |  |
| Gastrointestinal/hepatobiliary disease | 29 (6.0) | 1 (1.3) |  |
| Malignancies | 275 (56.6) | 5 (6.4) |  |
| Chronic neurological disease | 13 (2.7) | 3 (3.8) |  |
| Severity score at ICU admission |  |  |  |
| Initial SOFA score | 10.0 (7.0 – 12.8) | 7.0 (5.0 – 10.0) | <0.001 |
| Initial SAPS 3 | 62.0 (50.0 – 73.0) | 62.0 (62.0 – 62.0) | 0.682 |
| Primary diagnosis at ICU admission |  |  | <0.001 |
| Respiratory failure | 315 (64.8) | 9 (11.5) |  |
| Sepsis | 105 (21.6) | 5 (6.4) |  |
| Cardiovascular problems | 30 (6.2) | 64 (82.1) |  |
| Gastrointestinal/hepatobiliary problems | 28 (5.8) | 0 (0.0) |  |
| Neurologic problems | 3 (0.6) | 0 (0.0) |  |
| Others | 5 (1.0) | 0 (0.0) |  |
| Organ support during ICU stay |  |  |  |
| Continuous renal replacement therapy | 98 (20.2) | 24 (30.8) | 0.050 |
| Extracorporeal membrane oxygenation | 27 (5.6) | 30 (38.5) | <0.001 |
| Vasopressor | 330 (67.9) | 62 (79.5) | 0.054 |
| Clinical outcomes |  |  |  |
| Length of ICU stay, days | 10.2 (5.6 – 19.3) | 10.0 (4.7 – 19.0) | 0.594 |
| In-ICU mortality | 175 (36.0) | 6 (7.7) | <0.001 |

Data are presented as median and interquartile ranges or as numbers (%) of patients.

ICU, intensive care unit; SOFA, sequential organ failure assessment; SAPS, Simplified Acute Physiology Score; CAM-ICU, confusion assessment method for the intensive care unit; MV, mechanical ventilation.
