# Supplementary material for: Safety and feasibility of continuous ketamine infusion for analgosedation in medical and cardiac ICU patients who received mechanical ventilation support: A retrospective cohort study
Source: PLoS One. 2022 Sep 22;17(9):e0274865. doi: 10.1371/journal.pone.0274865 (PMC9499237; doi:10.1371/journal.pone.0274865)
Supplement: S2 Table — (DOCX) [file pone.0274865.s002.docx]

Supplementary Table 2. Pain, agitation, and sedation trends during continuous ketamine infusion according to clinical subgroups.

|  | 0~8 hr before ketamine infusion | 0~8 hr after ketamine infusion | 8~16 hr after ketamine infusion | 16~24 hr after ketamine infusion | P for trend |
| --- | --- | --- | --- | --- | --- |
| **Medical ICU (n=486)** |  |  |  |  |  |
| RASS |  |  |  |  |  |
| -2 ~ 0 | 109 (22.4) | 121 (24.9) | 123 (25.3) | 114 (23.5) | 0.497 |
| < -2 or > 0 | 144 (29.6) | 128 (26.3) | 116 (23.9) | 127 (26.1) | 0.174 |
| -4 and -5 | 233 (47.9) | 237 (48.8) | 247 (50.8) | 245 (50.4) | 0.174 |
| CAM-ICU |  |  |  |  |  |
| Positive | 200 (42.1) | 204 (42.0) | 192 (39.5) | 184 (37.9) | 0.174 |
| Negative | 286 (58.8) | 282 (58.0) | 294 (60.5) | 302 (62.1) | 0.174 |
| Critical care pain observation tool |  |  |  |  |  |
| 0.655 | 401 (88.7) | 436 (96.5) | 438 (96.9) | 391 (86.5) | 1.000 |
| Numeric rating scale |  |  |  |  |  |
| ≤ 3 | 21 (75.0) | 20 (71.4) | 20 (71.4) | 20 (71.4) | 0.180 |
| **Cardiac ICU (n=78)** |  |  |  |  |  |
| RASS |  |  |  |  |  |
| -2 ~ 0 | 18 (23.1) | 25 (32.1) | 21 (26.9) | 19 (24.4) | 1.000 |
| < -2 or > 0 | 41 (52.6) | 33 (42.3) | 36 (46.2) | 38 (48.7) | 1.000 |
| -4 and -5 | 19 (24.4) | 20 (25.6) | 21 (26.9) | 21 (26.9) | 0.071 |
| CAM-ICU |  |  |  |  |  |
| Positive | 36 (46.2) | 43 (55.1) | 36 (46.2) | 34 (43.6) | 0.279 |
| Negative | 42 (53.8) | 35 (44.9) | 42 (53.8) | 44 (56.4) | 0.279 |
| Critical care pain observation tool |  |  |  |  |  |
| ≤ 2 | 52 (69.3) | 67 (89.3) | 66 (88.0) | 66 (88.0) | 0.718 |
| Numeric rating scale |  |  |  |  |  |
| ≤ 3 | 0 (0) | 0 (0) | 0 (0) | 0 (0) | 1.000 |
| **Sepsis (n=110)** |  |  |  |  |  |
| RASS |  |  |  |  |  |
| -2 ~ 0 | 46 (41.8) | 43 (39.1) | 46 (41.8) | 33 (30.0) | 0.279 |
| < -2 or > 0 | 46. (41.8) | 44 (40.0) | 37 (33.6) | 51 (36.4) | 1.000 |
| -4 and -5 | 18 (16.4) | 23 (20.9) | 17 (15.5) | 26 (23.6) | 0.497 |
| CAM-ICU |  |  |  |  |  |
| Positive | 72 (65.5) | 75 (68.2) | 71 (64.5) | 66 (60.0) | 0.174 |
| Negative | 38 (34.5) | 35 (31.8) | 39 (35.5) | 44 (40.0) | 0.174 |
| Critical care pain observation tool |  |  |  |  |  |
| ≤ 2 | 88 (88.0) | 99 (99.0) | 99 (99.0) | 87 (87.0) | 0.718 |
| Numeric rating scale |  |  |  |  |  |
| ≤ 3 | 9 (100.0) | 9 (100.0) | 8 (88.9) | 8 (88.9) | 0.121 |
| **Other than sepsis (n=454)** |  |  |  |  |  |
| RASS |  |  |  |  |  |
| -2 ~ 0 | 149 (32.8) | 180 (39.6) | 184 (40.5) | 178 (39.2) | 0.497 |
| < -2 or > 0 | 223 (49.1) | 195 (43.0) | 183 (40.3) | 187 (41.2) | 0.174 |
| -4 and -5 | 82 (18.1) | 79 (17.4) | 83 (8.3) | 89 (19.6) | 0.174 |
| CAM-ICU |  |  |  |  |  |
| Positive | 227 (50.0) | 213 (46.9) | 255 (56.2) | 248 (54.6) | 0.497 |
| Negative | 227 (50.0) | 241 (53.1) | 199 (43.8) | 206 (45.4) | 0.497 |
| Critical care pain observation tool |  |  |  |  |  |
| ≤ 2 | 365 (85.5) | 404 (94.6) | 405 (94.8) | 370 (86.7) | 0.497 |
| Numeric rating scale |  |  |  |  |  |
| ≤ 3 | 18 (85.7) | 17 (81.0) | 19 (90.5) | 18 (85.7) | 0.718 |
| **Shock^*^ (n=392)** |  |  |  |  |  |
| RASS |  |  |  |  |  |
| -2 ~ 0 | 127 (23.4) | 146 (37.2) | 144 (36.7) | 133 (33.9) | 1.000 |
| < -2 or > 0 | 185 (47.2) | 162 (41.3) | 152 (38.8) | 165 (42.1) | 0.497 |
| -4 and -5 | 80 (20.4) | 84 (21.4) | 96 (24.5) | 94 (24.0) | 0.174 |
| CAM-ICU |  |  |  |  |  |
| Positive | 236 (60.2) | 247 (63.0) | 228 (58.2) | 218 (55.6) | 0.174 |
| Negative | 156 (39.8) | 145 (37.0) | 164 (41.8) | 174 (44.4) | 0.174 |
| Critical care pain observation tool |  |  |  |  |  |
| ≤ 2 | 314 (85.8) | 351 (95.9) | 344 (94.0) | 321 (87.7) | 1.000 |
| Numeric rating scale |  |  |  |  |  |
| ≤ 3 | 17 (89.5) | 17 (89.5) | 16 (84.2) | 16 (84.2) | 0.121 |
| **Without shock (n=172)** |  |  |  |  |  |
| RASS |  |  |  |  |  |
| -2 ~ 0 | 68 (39.5) | 77 (44.8) | 86 (50.0) | 78 (45.3) | 0.174 |
| < -2 or > 0 | 84 (48.8) | 78 (45.3) | 68 (39.5) | 73 (42.4) | 0.174 |
| -4 and -5 | 20 (11.6) | 17 (9.9) | 18 (10.5) | 21 (12.2) | 0.497 |
| CAM-ICU |  |  |  |  |  |
| Positive | 113 (65.7) | 116 (67.4) | 98 (57.0) | 96 (55.8) | 0.174 |
| Negative | 58 (33.7) | 56 (32.6) | 74 (43.0) | 76 (44.2) | 0.174 |
| Critical care pain observation tool |  |  |  |  |  |
| ≤ 2 | 139 (86.3) | 152 (94.4) | 146 (90.7) | 122 (75.8) | 0.497 |
| Numeric rating scale |  |  |  |  |  |
| ≤ 3 | 6 (54.5) | 4 (36.4) | 6 (54.5) | 6 (54.5) | 0.655 |

Data are presented as numbers (%) of patients.

^*^Shock is defined as the patients who supported vasopressors or inotropes.

RASS, Richmond agitation sedation scale; CAM-ICU, confusion assessment method for the intensive care unit.
